# Interactions of Silicon With Essential and Beneficial Elements in Plants

**DOI:** 10.3389/fpls.2021.697592

**Published:** 2021-06-23

**Authors:** Jelena Pavlovic, Ljiljana Kostic, Predrag Bosnic, Ernest A. Kirkby, Miroslav Nikolic

**Affiliations:** ^1^Institute for Multidisciplinary Research, University of Belgrade, Belgrade, Serbia; ^2^Faculty of Biological Sciences, Leeds University, Leeds, United Kingdom

**Keywords:** silicon, nutrients, beneficial elements, deficiency, toxicity, transporters

## Abstract

Silicon (Si) is not classified as an essential element for plants, but numerous studies have demonstrated its beneficial effects in a variety of species and environmental conditions, including low nutrient availability. Application of Si shows the potential to increase nutrient availability in the rhizosphere and root uptake through complex mechanisms, which still remain unclear. Silicon-mediated transcriptional regulation of element transporters for both root acquisition and tissue homeostasis has recently been suggested as an important strategy, varying in detail depending on plant species and nutritional status. Here, we summarize evidence of Si-mediated acquisition, uptake and translocation of nutrients: nitrogen (N), phosphorus (P), potassium (K), calcium (Ca), magnesium (Mg), sulfur (S), iron (Fe), zinc (Zn), manganese (Mn), copper (Cu), boron (B), chlorine (Cl), and nickel (Ni) under both deficiency and excess conditions. In addition, we discuss interactions of Si-with beneficial elements: aluminum (Al), sodium (Na), and selenium (Se). This review also highlights further research needed to improve understanding of Si-mediated acquisition and utilization of nutrients and *vice versa* nutrient status-mediated Si acquisition and transport, both processes which are of high importance for agronomic practice (e.g., reduced use of fertilizers and pesticides).

## Introduction

Silicon (Si) is the second most abundant element (after oxygen) in the Earth’s crust, mainly occurring as various silicate minerals in most soils. The concentration of the plant-available form of Si in soil solutions, monosilicic acid (H_4_SiO_4_), varies between 0.1 and 0.6 mM, which is about two orders of magnitude higher than the concentrations of phosphorus (P) (Yan et al., 2018). Silicon in taken up and translocated through the plant to be deposited as SiO_2_ phytoliths in the lumen, cell walls and intercellular spaces (see Hodson and Evans, 2020). However, plants species differ greatly in their ability to accumulate Si with values ranging from 0.1% to 10% Si on a dry weight basis (Epstein, 1994, 1999). Consequently, some plant species are minimally affected by Si fertilization compared to others (Coskun et al., 2019). This variation among species can be explained by different expression and functionality of Si transporters (see “Essential and Beneficial Element Status Affects Silicon Uptake and Distribution” section, below).

The essentiality of Si for plants has been the matter of a long debate dating back to the 19th century (e.g., J. von Sachs *versus* E. Wolff and C. Kreuzhage, see Sreenivasan, 1934; Katz et al., 2021). It is now reasonably well established that Si is essential for only a few species of plants, the silicophiles, high in Si concentration (see Marschner, 2012; Hodson and Evans, 2020). However, its classification as a beneficial element has been recognized for more than 50 years by those teaching and researching in plant nutrition (e.g., Mengel and Kirkby, 2001; Epstein and Bloom, 2008; Marschner, 2012). Numerous more recent studies, have confirmed its beneficial effects in a variety of species growing under a wide range of environmental conditions as reviewed by, e.g., Epstein (1994, 1999), Ma (2004); Liang et al. (2007) and Debona et al. (2017). This overwhelming evidence together with studies of Si transporters in plants and yield benefits of Si fertilization of crops probably eventually led the International Plant Nutrition Institute (IPNI) to upgrade Si from complete omission to a listing as “beneficial substance” in 2015 (Coskun et al., 2019). Indeed, as far as we are aware, Si appears to be the only known element that effectively alleviates both biotic (pathogens and pests) and abiotic (e.g., drought, salinity, heavy metals, UV irradiation, nutrient imbalance) stresses in many plant species. On the other hand, its physiologically versatile mode of action, regardless of the stress, is still uncertain and has recently been debated by Coskun et al. (2019). Interestingly, the ability of Si to alleviate different stresses has been confirmed even in species with a low Si accumulation potential, such as tomato (Hoffmann et al., 2020; Sun et al., 2020).

**FIGURE 1:**
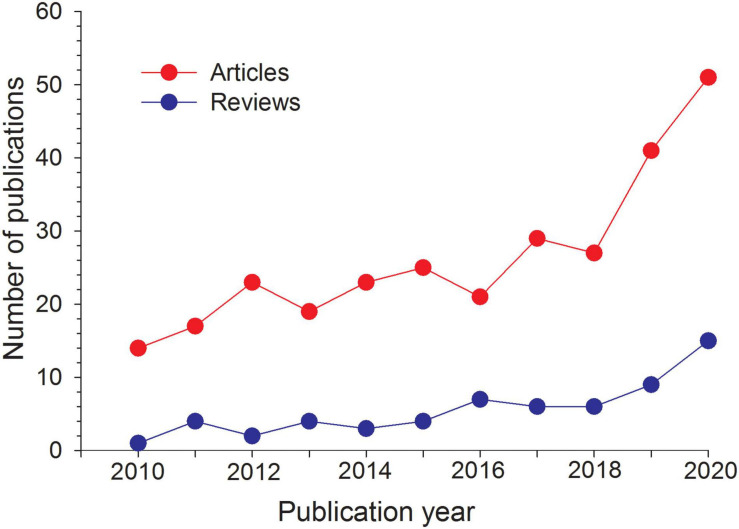
Number of publications related to silicon in plants from 2010 to 2020, based on Scopus search with the title words: “Silicon” or “Silicate” and “Plants,” refined to “Agricultural and Biological Sciences” (March, 2021). Total number of publications: 351; research articles: 290; reviews: 61.

The past decade has seen a considerable increase in knowledge of the role of Si in plant biology and agriculture, as illustrated by the rapidly increasing number of both research and review articles (Figure 1), including recent special topics or issues devoted to Si in plants in peer-reviewed international journals (e.g., *Frontiers in Plant Science*, *Journal of Experimental Botany*, *Plants*). In general, most of the recent Si-related reviews deal with cycling and mobility of Si in soil, transport of Si (including Si transporters) in plants and on its role in biotic and/or abiotic stresses, while only a few focus on the role of Si on alleviating deficiency of nutrients (Herandez-Apaolaza, 2014; Ali et al., 2020).

This review attempts to summarize current evidence of interactions of Si with essential (nutrients) and beneficial elements in various crop species. We consider not only the ways in which Si supply affects rhizosphere mobility, uptake and transport of nutrient elements under different environmental conditions, but also the influence of plant nutrient status on the acquisition and transport of Si itself. Here we have not focused on Si chemistry and interactions of Si with other elements in soil mainly because this issue has already been updated in the review of Schaller et al. (2021) and also because of lack of evidence for direct mobilization of elements by Si in agricultural soils, with exception of peatlands (Reithmaier et al., 2017; Hömberg et al., 2020). After much deliberation we decided to base this review on individual elements rather than processes (e.g., rhizosphere acquisition, uptake, transport, utilization), taking the view that this approach would be more conducive to the reader and to finding information of interest. Finally, by comparative analysis of the literature describing both direct and indirect effects of Si in nutrient acquisition and utilization, we suggest research directions to elucidate how Si functions to support plants to overcome nutrient and beneficial element disorders, processes which urgently need to be explored experimentally and their implications for agricultural practice advanced.

## Interactions of Silicon With Macronutrients

### Nitrogen

Application of Si positively affects almost all aspects of nitrogen (N) nutrition, i.e., uptake, assimilation, and remobilization (Detmann et al., 2012; Wu et al., 2017; Haddad et al., 2018; Gou et al., 2020). This beneficial effect of Si on overall plant performance has been established under low, optimal, and excessive N supply. In addition to a direct effect on N metabolism, Si supply induces changes in carbon (C) and P stoichiometry in shoots (Schaller et al., 2012; Neu et al., 2017; Deus et al., 2020) and increases nutrient acquisition by roots (Barreto et al., 2017), which in turn can contribute to improving N utilization within plant tissues. Plant species and genotypes preferentially utilize different mineral N sources (NO_3_^–^ or NH_4_^+^), and show substantial variation in strategies to cope with N imbalance (Esteban et al., 2016). This may explain the high variation in interactions between Si and N observed in different plant species.

Increased N uptake under suboptimal N supply mediated by Si has been reported in different plant species, e.g., cowpea (*Vigna unguiculata*) (Mali and Aery, 2008a), maize (*Zea mays*) (Mabagala et al., 2020), rice (*Oryza sativa*) (Pati et al., 2016; Cuong et al., 2017; Deus et al., 2020). Increased tissue concentration of N has been attributed to enhanced N_2_ fixation (Mali and Aery, 2008a), increased N availability in soil, transcriptional up-regulation of genes involved in N uptake (Haddad et al., 2018; see Table 1), and enhanced transport efficiency (Sheng et al., 2018). Up-regulation of genes encoding NO_3_^–^ transporters (*BnaNTR2*.*1* and *BnaNTR1.1*) was proposed as an important strategy to overcome N deficiency in rapeseed (*Brassica napus*). Moreover, Si supplementation increased expression of *BnaNTR2.1* even before imposition of N starvation. In addition to the effect on gene expression, it has been suggested that Si increases the efficiency of the NH_4_^+^ transporter (OsAMT) without up-regulation of *OsAMT* (Sheng et al., 2018). On the other hand, enhanced rate of N assimilation has been proposed as another Si-mediated mechanism to alleviate N starvation (Wu et al., 2017). In rice grown at low N supply, the relative expressions of N-uptake genes (*OsNTR1.1* and *OsAMT1;1*) were unaffected or even down-regulated by Si supply, whereas N-assimilation genes (*OsGS2*, *OsFd-GOGAT*, *OsNADH-GOGAT2*, *OsGDH2*, and *OsNR1*) were up-regulated (Wu et al., 2017). Even in non-stressed rice with an adequate N supply, Si nutrition was shown to modulate the flux from 2-oxoglutarate into amino acid metabolism (Detmann et al., 2012). However, the exact mechanisms by which Si induces change in N metabolism still remain unknown. One explanation could be that Si mediates increase in cytokinin activity in the leaf N source/sink balance via the increased expression of the gene *CWINV1* encoding β-fructofuranosidase, insoluble isoenzyme 1 (Markovich et al., 2017).

**TABLE 1:** Evidence for Si-mediated expression of genes encoding transporters for essential and beneficial elements in plants.

Conditions	Plant species	Localization/direction/form/function	Transporter genes	Expression pattern by Si	References
Low N	Rapeseed	Root/influx/nitrate/uptake	*BnNRT1.1*,	Up-regulated	Haddad et al., 2018
			*BnNRT2.1*		
High N	Rice	Root/influx/ammonium/uptake	*OsAMT1;1*	Down-regulated	Wu et al., 2017
		Root/influx/nitrate/uptake	*OsNTR1.1*		
Low P	Wheat	Root/influx/phosphate/uptake	*TaPHT1;1*,	Up-regulated	Kostic et al., 2017
			*TaPHT1;2*		
High P	Rice	Root/influx/phosphate/uptake	*OsPHT1;6*	Down-regulated	Hu et al., 2018
Low K	Sorghum	Root cortex/influx/K^+^/xylem unloading	*SbHAK5, SbAKT1*	Down-regulated	Chen et al., 2016b
		Root cortex/efflux/K^+^/xylem loading	*SbSKOR1*,	Up-regulated	
			*SbSKOR2*		
Low S	Rice	Root/influx/sulfate/uptake	*OsSULTR1;1*	Down-regulated (up to 3 d of S deficiency)	Réthoré et al., 2020
			*OsSULTR1;2*	Down-regulated	
			*OsSULTR2;1*	Down-regulated (from 1 to 3 d of S deficiency)	
			*OsSULTR2;2*	Down-regulated (15 d of S deficiency)	
Low Fe	Cucumber	Root/influx/Fe^2+^/uptake	*CsIRT1*	Up-regulated (3 d of Si supply)	Pavlovic et al., 2013
		Leaf/efflux/Fe(II)-complex/phloem mobilization	*CsYSL1*	Up-regulated	Pavlovic et al., 2016
	Barley	Root/influx/Fe(III)-complex/uptake	*HvYS1*	Up-regulated (5 h of Si supply)	Nikolic et al., 2019
High Mn	Rice	Root/influx/Mn^2+^/uptake	*OsNramp5*	Down-regulated	Che et al., 2016
High Cu	Arabidopsis	Root/influx/Cu^2+^/uptake	*AtCOPT1*,	Down-regulated	Li J. et al., 2008
			*AtHMA5*		
	Rice	Root/influx/metal ions/translocation	*OsHMA2*	Down-regulated	Kim et al., 2014
		Root/efflux/metal ions/sequestration	*OsHMA3*	Down-regulated	
	Tobacco	Root/influx/Cu^2+^/uptake	*NtCOPT1*	Down-regulated	Flora et al., 2019
High Zn	Rice	Root/influx/Zn^2+^/uptake	*OsZIP1*	Down-regulated	Huang and Ma, 2020
High B	Barley	Leaf/efflux/H_3_BO_3_/export out of symplast	*HvBOR2*	Up-regulated	Akcay and Erkan, 2016
High Na	Maize	Root/efflux/Na^+^/export and xylem loading	*ZmSOS1, ZmSOS2*	Up-regulated	Bosnic et al., 2018
		Root/influx/Na^+^/uptake	*ZmHKT1*	Down-regulated	
		Leaf/influx/Na^+^/vacuolar sequestration	*ZmNHX*	Up-regulated	

Silicon fertilization is known to improve N use efficiency and agronomic parameters of crops (yield and nutritional value) as observed in the long-term experiments in rice (Cuong et al., 2017), maize (Mabagala et al., 2020), rapeseed (Laine et al., 2019), and wheat (*Triticum aestivum*) (Neu et al., 2017). Although the underlying mechanism has not been studied in detail, it has been demonstrated that Si-induced biosynthesis of amino acids and remobilization of N (Detmann et al., 2012) can partially explain this phenomenon. Theoretically, remobilization of N (mainly in amino-acids forms) from storage pools in vegetative organs to the grains is considered as one of the major factors regulating high seed yields (Tegeder and Masclaux-Daubresse, 2017). Another proposed effect of Si might be alteration of C:N:P stoichiometry (Schaller et al., 2012; Neu et al., 2017; Deus et al., 2020). The science underpinning these studies is that in Si accumulator plants especially (e.g., rice, grasses and sugarcane), Si might partially replace C in shoot tissues to stabilize function and enhance photosynthesis thereby changing C:N:P stoichiometry. The highly beneficial effects of partial replacement of C by Si have recently been shown very clearly in a pot experiment with soil applied Si to sugarcane (Frazāo et al., 2020). Replacement of C by Si induced lower plant C concentration and was associated with greater biomass production resulting from higher rates of N and P uptake and accumulation and enhanced rates of photosynthesis. Considering that Si incorporation into vegetative tissues is bioenergetically cheaper than the formation of structural C compounds, plants might well benefit from altered C:N stoichiometry, especially under limited N supply (Neu et al., 2017).

Enhanced growth of Si-fed plants exposed to high N concentration was often attributed to increased photosynthetic efficiency, antioxidant capacity and improved water status (Barreto et al., 2017; Viciedo et al., 2019; Campos et al., 2020). However, responses to Si application differ even among cultivars of the same species (Barreto et al., 2017, 2021; Campos et al., 2020). In particular, supply of Si is of importance for the high yielding cultivation systems with dense planting and large application doses of N fertilizers (Liang et al., 2015). Under such conditions, excess N causes lodging, mutual shading and susceptibility to biotic stresses. Recent evidence suggests a new role of Si in reducing N uptake (Wu et al., 2017), optimization of N assimilation (Gou et al., 2020), or increased uptake of cations (Barreto et al., 2017) under high N supply. In rice exposed to high N supply, Si primarily affected N uptake genes (*OsNTR1.1* and *OsAMT1;1*), reducing their expression level and therefore limiting N uptake (Wu et al., 2017). Apart from *OsGS1*, none of the genes involved in N assimilation were affected by Si nutrition under N excess (Wu et al., 2017). A possible explanation for the down-regulation of N-uptake genes by Si is increased cytokinin level (Kiba et al., 2011; Barreto et al., 2021), however, this proposal needs to be supported by further experimental evidence. By contrast, increased activity of N-assimilation enzymes (NR, NiR, GS and GOGAT, and NADH-GDH) was suggested as a key factor of Si-mediated alleviation of nitrate toxicity in cucumber (*Cucumis sativus*) cv. Jinyou 1 (Gou et al., 2020). Supply of Si in cauliflower (*Brassica oleracea var. Botrytis*) and broccoli (*Brassica oleracea var. Italica*) exposed to NH_4_^+^ toxicity increased uptake of K^+^ (Barreto et al., 2017), which activates enzymes GS and GDH that convert ammonium to amino acids and therefore mitigate tissue accumulation of toxic NH_4_^+^ (Sarasketa et al., 2014; Esteban et al., 2016).

### Phosphorus

The influence of Si on P nutrition has been extensively studied. Evidence suggests that Si plays a significant role in P nutrition, but the precise nature of that role still remains unclear; in particular, experimental evidence at molecular or protein level is scarce. So far, an alleviating effect of Si under P limiting conditions has been reported in wheat (Kostic et al., 2017; Neu et al., 2017), maize (Owino-Gerroh and Gascho, 2005), tomato (*Solanum lycopersicum*) (Zhang et al., 2019), rice (Ma and Takahashi, 1990; Pati et al., 2016; Hu et al., 2018) and potato (*Solanum tuberosum*) (Soltani et al., 2017; Soratto et al., 2019). Two major mechanisms of Si-mediated alleviation of P deficiency are proposed: (1) increased root uptake and (2) enhanced utilization of P within the plant tissues. Increased P uptake followed by soil Si fertilization has widely been reported (Owino-Gerroh and Gascho, 2005; Neu et al., 2017; Zhang et al., 2019). Silicon can increase P availability in soil, through pH changes, decreased P sorption by soil minerals, due to competition between P and Si (as depending on Si speciation in soil solution) or by changes in the dynamics of the microbial community (e.g., P-mobilizing microorganisms) (Owino-Gerroh and Gascho, 2005; Li et al., 2019; Schaller et al., 2019, 2021). However, the effect of Si on soil availability of P is dependent on soil type, mineralogical and microbial composition, pH, as well as on type and amount of Si fertilizers (Hu et al., 2021). In addition, some Si fertilizers increase soil pH and thus eradicate the rhizotoxic effect of Al^3+^ in acid low P soils, thereby restoring overall root acquisition potential (Kostic et al., 2017).

On the other hand, the effect of Si on increased gene expression relating to inorganic P (Pi) uptake has been demonstrated in wheat (Kostic et al., 2017). In an acid low P soil, Si up-regulated genes encoding Pi transporters (*TaPHT1;1* and *TaPHT1;2*) and consequently increased Pi uptake. The effect of Si on up-regulation of transcript levels of Pi transporters was significantly greater than the effect of liming, suggesting that the ameliorative Si effect could not simply be attributed to Si-induced pH correction and increased P availability in soil. Silicon also stimulated root Pi acquisition by increasing the exudation of carboxylates (Kostic et al., 2017), although the effect of exuded carboxylates on P-mobilization in the soil is still controversial (Wang et al., 2016).

Improved internal P utilization as a response to Si supplementation in a low P environment was observed in rice and potato (Ma and Takahashi, 1990; Soratto et al., 2019). In rice, Si application resulted in decreased Fe and Mn uptake, while P uptake was unaffected (Ma and Takahashi, 1990). Given the high affinity of phosphates to metals such as Fe and Mn, the internal availability of P in plants may be affected by levels of these metals when P concentration is low (Ma and Takahashi, 1990). The influence of Si on Fe and Mn uptake is discussed in detail in “Interactions of Silicon with Micronutrients” section, below. Results for Si application to potato on a soil low in P were similar to those for rice. Si did not increase total P uptake, but concentrations of soluble inorganic P and total soluble P of the leaves were enhanced (Soratto et al., 2019). Soluble inorganic P is the main plant P reserve and it is tightly regulated. Plants have developed a series of coordinated responses to conserve, recycle, and remobilize internal inorganic P to maintain P homeostasis (Chiou and Lin, 2011). The increase in soluble inorganic P concentration in leaves as a response to Si application therefore indicates that Si may play a role in maintenance of plant growth under low P availability (Soratto et al., 2019).

Excessive P levels in soil disturb plant growth and reduce food quality (Zhang et al., 2015; Nikolic et al., 2016). The Si-mediated decrease in P uptake under excessive P supply has been observed in several plant species such as rice (Hu et al., 2018), soybean (*Glycine max*) (Miyake and Takahashi, 1985), strawberry (*Fragaria ananassa*) (Miyake and Takahashi, 1986), and cucumber (Marschner et al., 1990). This decrease is often attributed to the formation of physical apoplastic barriers by Si deposition in the root thereby decreasing P uptake (Ma, 2004). The effect of Si in regulating root Pi transporters was demonstrated, for the first time in rice by Hu et al. (2018). Under excessive P supply, Si amendment decreased expressions level of *OsPHT1;6* and consequently decreased Pi uptake. The importance of Si accumulation in shoots on P uptake was elegantly demonstrated by comparing expression levels of *OsPHT1;6* and P accumulation between wild type (WT) rice cultivar Oochikara and a rice mutant *lsi1* defective in the Lsi1 transporter for Si uptake (Hu et al., 2018).

### Potassium

The interaction of Si with potassium (K) in plants has been less well investigated than that with N and P, although many studies have reported that Si can affect tissue K concentration under stress conditions such as salinity, drought or N excess (Kaya et al., 2006; Barreto et al., 2017; Yan et al., 2021b). Over the past decade, an increasing number of studies have focused on the direct effect of Si on K-deprived plants (Miao et al., 2010; Chen et al., 2016a,b; Hosseini et al., 2017; Buchelt et al., 2020; dos Santos Sarah et al., 2021). Increased uptake of K and restored physiological performance impaired by K deficiency has been reported as the main beneficial role of Si in K-deficient plants. In soybean and some forage crops (*Panicum maximum* and *Brachiaria ruziziensis × Brachiaria brizanth*), Si supplementation resulted in higher K concentration in leaves (Miao et al., 2010; Buchelt et al., 2020). In addition, Si also alleviated K-deficiency-induced membrane lipid peroxidation and oxidative stress by modulating antioxidant enzymes (Miao et al., 2010; Chen et al., 2016a).

Increased K uptake by Si supplied rice plants grown under saline conditions may possibly be explained by Si-mediated stimulation of H^+^-ATPase activity (Liang et al., 2003) or up-regulation of K^+^ transporter genes, i.e., *OsHAK5*, *OsAKT1*, and *OsSKOR* by Si (Yan et al., 2021b). On the other hand, in K-deprived maize and sorghum, Si supplementation did not increase K uptake but restored physiological activity commonly impaired by K deficit, i.e., water use efficiency and photosynthesis (Chen et al., 2016b; dos Santos Sarah et al., 2021). High xylem K^+^ concentration reduces osmotic potential and thus contributes to xylem hydraulic conductance (Zwieniecki et al., 2001; Nardini et al., 2010). Chen et al. (2016b), showed that increased K^+^ concentration in the xylem sap of Si-fed plants was accompanied by up-regulated transcript levels of *SKOR* genes (*SKOR1* and *SKOR2*) mediating K secretion from root cortex cells into the xylem and down-regulated transcript levels of *AKT1* and *HAK5* (Table 1) to maintain high K in the xylem. The authors propose that Si plays a role in plant signaling regulation under K-deficient stress, but clear evidence of this is still lacking. In relation to drought stress, Hosseini et al. (2017) investigated the effects of combined K deficiency and PEG-induced osmotic stress in barley (*Hordeum vulgare*). Since Si did not did not have a direct ameliorative effect on K deficiency, the authors suggest that the beneficial role of Si appears most likely mediated by abscisic acid (ABA) homeostasis and increased activity of cytokinin isopentenyl adenine.

### Calcium

Different effects of Si on calcium (Ca) uptake and accumulation are reported in the literature depending on plant species, experimental conditions, stress type, and amount of Si applied. Several studies have reported that Si promotes Ca uptake in various crops grown under optimal conditions (Mali and Aery, 2008b; Gottardi et al., 2012; Greger et al., 2018), or in plants exposed to various forms of stress, as for instance in maize grown under drought stress (Kaya et al., 2006). By contrast, other studies suggested that Si does not affect (Cooke and Leishman, 2016) or even decreases Ca accumulation (Ma and Takahashi, 1993; Brackhage et al., 2013; Jang et al., 2018). Moreover, a progressive decline in tissue Ca accumulation with increasing Si supply has been demonstrated in rice and common reed (*Phragmites australis*) (Brackhage et al., 2013; Jang et al., 2018). However, these studies were not directly focused on the Si effect in Ca-deficient plants. Decreased Ca accumulation in response to Si application can be attributed to lowered transpiration caused by deposition of Si in the leaves (Ma and Takahashi, 1993), reduced Ca^2+^ uptake due to biosilicification of root structures (Fleck et al., 2015) and Si-Ca interaction in the growing media or apoplast (Dishon et al., 2011). Recently, Bosnic et al. (2019c) reported that high Si supply may also decrease activity of Ca^2+^ in nutrient solution high in Na^+^ under alkaline conditions. Increased Ca uptake can result as a consequence of Si-mediated alleviation of primary stress, due to restored plasma membrane integrity and increased activity of H^+^-ATPase (Liang, 1999; Kaya et al., 2006). Experimental evidence of a direct role of Si in Ca-deficient plants is still lacking. Very recently da Silva et al. (2021) demonstrated Si-mediated alleviation of Ca deficiency in cabbage (*Brassica oleracea var. capitata*) grown in water culture which revealed an interesting Ca-Si interaction pattern. In Ca-sufficient cabbage, Si addition decreased Ca accumulation in aerial parts but increased it in roots whereas in Ca-deficient cabbage, Si addition increased Ca accumulation in aerial parts but was without effect on root accumulation.

### Magnesium and Sulfur

According to the limited available literature on interactions of Si with magnesium (Mg), the effect of Si appears to depend on plant species and environmental circumstances. Greger et al. (2018) reported that the addition of Si increased Mg uptake and accumulation in the shoots of several species grown in solution culture with optimal nutrient supply. Other studies likewise reported Si-mediated Mg accumulation in plants exposed to stress, as for instance under ammonium excesses in broccoli and cauliflower (Barreto et al., 2017) and in P-deficient tomato (Zhang et al., 2019). Investigating Si supply to drought stressed cultivars of sunflower (*Helianthus annuus*) in a pot experiment, 8 of the 12 cultivars accumulated higher amounts of Mg in their shoots when supplied with Si compared to drought stress alone (Gunes et al., 2009). The effect on uptake *per se*, however, was not so clear-cut when yield data were taken into account (Gunes et al., 2008). To date, the effect of Si on the expression of Mg transporters has not been demonstrated. Indeed, only two studies have investigated the effect of Si on Mg-deficient plants. Buchelt et al. (2020) reported Si-mediated alleviation of Mg stress in forage crops, but attributed it to increased Mg use efficiency, rather than increased Mg uptake. The study by Hosseini et al. (2019) showed that Si supply had no influence on the uptake and/or translocation of Mg in maize plants. The authors proposed the growth of the deficient plants was sustained indirectly by the beneficial role of Si in significantly increasing the levels of chlorophyll and by regulating sugar metabolism and hormonal balance (Hosseini et al., 2019).

Work on interactions of Si with sulfur (S) is at a preliminary stage. Early results indicate that uptake and accumulation of S was unaffected by Si supply in forages crops (*Panicum maximum* and *Brachiaria ruziziensis* × *Brachiaria brizantha*) (Buchelt et al., 2020) while Si supply even decreased shoot accumulation of S in barley and rice exposed to S deficiency (Maillard et al., 2018; Réthoré et al., 2020). Molecular analysis also showed that the addition of Si tended to decrease transcript levels of S transporters (OsSULTR) in rice (Réthoré et al., 2020; see Table 1).

## Interactions of Silicon With Micronutrients

### Iron

The effect of Si on iron (Fe) nutrition was clearly demonstrated in different plant species grown in optimal, low or high Fe conditions (e.g., Gonzalo et al., 2013; Pavlovic et al., 2013; Chalmardi et al., 2014; dos Santos et al., 2019; Nikolic et al., 2019; Becker et al., 2020; Hernández-Apaolaza et al., 2020). In general, the results suggest that Si addition strongly affects Fe availability in the rhizosphere and the root apoplast (directly or indirectly), as well as the expression of genes involved in Fe transport at both root and leaf level, thus influencing Fe uptake, translocation and distribution within different plant organs and tissues.

The alleviating effect of Si on Fe deficiency has been shown in both Strategy 1 (dicots and non-graminaceous monocots with reduction-based Fe uptake; Gonzalo et al., 2013; Pavlovic et al., 2013; Bityutskii et al., 2014) and Strategy 2 (graminaceous monocots that exhibit chelation-based Fe uptake; Nikolic et al., 2019; Teixeira et al., 2020) plant species. Moreover, the Si-ameliorative effect on Fe deficiency was found to be species-specific and pH-dependent (Gonzalo et al., 2013; Bityutskii et al., 2018). Supplying Si to cucumber roots, extended the binding pool for Fe in the root apoplast, and further promoted apoplastic Fe-mobilization by increasing the expression of key genes involved in the biosynthesis of organic acids acting as strong Fe chelators, i.e., *ICD* for citric acid and *MDH* for malic acid (Pavlovic et al., 2013). Silicon supply also induces up-regulation of the Strategy 1 genes (*FRO* encoding transmembrane protein involved in Fe^3+^ reduction and *IRT1* encoding Fe^2+^ transporter; see Table 1) in both common corn salad (*Valerianella locusta*) (Gottardi et al., 2012) and cucumber (Pavlovic et al., 2013). Enhancement of Fe distribution toward apical shoot parts, along with the tissue accumulation of Fe-mobilizing compounds such as citrate (in leaves and roots) and catechin (in roots) appears to be the major alleviating effect of Si (Bityutskii et al., 2014). Furthermore, Stevic et al. (2016) showed that addition of Si(OH)_4_ to Fe-deprived cucumber plants can increase Fe bioavailability through formation of an Fe-Si complex and maintaining the redox potential in both root apoplastic and xylem fluids, thus facilitating root-to-shoot Fe translocation via the xylem. At the leaf level, Si stimulated the expression of both *CsNAS1*, and subsequent accumulation of nicotianamine (NA), and Cs*YSL1* the encoding transporter involved in phloem loading/unloading of the Fe–NA complex, thereby enhancing remobilization and retranslocation of Fe from older (sink) to younger (source) leaves in Fe-deficient cucumber plants (Pavlovic et al., 2016; Table 1). Moreover, Nikolic et al. (2019) recently reported that Si mitigates Fe deficiency in barley (Strategy 2 species) by enhancing the expression of genes involved in Fe uptake and transport in roots, such as *HvNAS1* and *HvDMAS1* [responsible for biosynthesis of phytosiderophores (PS)], *HvTOM1* (encoding efflux transporter of PS), and *HvYS1* (encoding influx transporter for Fe-PS), thereby increasing Fe uptake. Additionally, Si supply increased the transcript abundance of *HvYS1* and *HvDMAS1* in leaves, responsible for metal redistribution between root and shoot. Interestingly, the effect of Si on regulation of both Strategy 1 and Strategy 2 genes transcripts showed strong time-dependency (see Table 1).

The role of Si in Fe-toxicity, extensively studied in rice, demonstrated that Si alleviates Fe-toxicity through precipitation of Fe in the growth media or formation of Fe plaque at the root surface (Fu et al., 2012). It has been suggested that Si supply decreases Fe uptake and translocation to aerial parts in rice, thus lowering Fe concentrations in both leaf and root tissues of plants exposed to Fe excess (Chalmardi et al., 2014; Dufey et al., 2014; dos Santos et al., 2019). Several recent studies on rice (Carrasco-Gil et al., 2018.; Becker et al., 2020) and cucumber (Hernández-Apaolaza et al., 2020) have reported that the addition of Si to the growth media caused higher Fe plaque formation, thus decreasing Fe uptake and activating root Fe deficiency responses even at optimal Fe supply, supporting the hypothesis postulated by Coskun et al. (2019) that Si may cause an apoplastic obstruction.

### Zinc

Several strands of evidence indicate interaction between Si and zinc (Zn) in plants under both deficient and excess Zn conditions (e.g., Gu et al., 2012; Bityutskii et al., 2014). For instance, Si application prevented certain symptoms of Zn-deficiency (necrotic spots) in cucumber plants, most probably due to its indirect effect by enhancing antioxidant defense capacity in plant tissues, rather than to its direct effect on mobility, uptake and tissue distribution of Zn (Bityutskii et al., 2014). In rice, Si supply increased shoot biomass and grain yield under low Zn conditions, which was associated with increased Zn concentration in the shoots (Mehrabanjoubani et al., 2015). Pascual et al. (2016) proposed that Si treatment enhanced Zn accumulation in the root apoplast as well as its movement to shoots when soybean plants were subjected to Zn deficiency, thus mitigating stress symptoms. However, the direct effects of Si on Zn-transporters by maintaining optimum Zn-concentration within plants have not as yet been verified.

The beneficial effects of Si have been observed under Zn-toxicity stress in several crop species such as rice (Song et al., 2011, 2014; Gu et al., 2012; Huang and Ma, 2020), cotton (*Gossypium hirsutum*) (Anwaar et al., 2015) and maize (Vieira da Cunha et al., 2008; Kaya et al., 2009). However, in some studies Si application did not alleviate Zn-toxicity as for instance in young seedlings of sorghum (Masarovič et al., 2012) and maize (Bokor et al., 2013). In spring sandwort (*Minuartia verna*), a dicotyledonous Si-accumulator, co-precipitated Zn-silicates were detected in the leaf epidermal cell wall which was associated with Zn tolerance (Neumann et al., 1997). Gu et al. (2012) also observed Zn and Si co-localized in cell walls in stems, sheaths and leaves of rice seedlings after Si addition. In addition to *in planta* Zn-Si colocalization, many studies reported that Si reduces bioavailability of Zn in soil by allocating this metal into more stable fractions such as organic matter and crystalline Fe-oxides (Vieira da Cunha et al., 2008). Fan et al. (2016) showed that Si affects the exudation of various organic acids (e.g., oxalic, acetic, tartaric, maleic and fumaric acids), from rice roots which may be involved in amelioration of Zn toxicity possibly by immobilization/co-precipitation in the soil solution.

Song et al. (2011) demonstrated that Si supply significantly decreased Zn concentration in shoots of two rice cultivars differing in tolerance to Zn excess, indicating lower root-to-shoot translocation of Zn, despite increased root Zn concentrations. However, the authors suggest that Si-mediated alleviation of Zn toxicity is mainly attributed to increased Si-mediated antioxidant defense capacity and membrane integrity (Song et al., 2011). Furthermore, Huang and Ma (2020) investigated the interaction between Si and Zn in rice at different levels of Zn supply using WT cv. Oochikara and its *lsi1* mutant defective in Si uptake. In contrast to the previous study (Song et al., 2011), a short-term uptake experiment of Huang and Ma (2020) demonstrated that Si decreased root uptake of the stable isotope ^67^Zn, but did not affect the root-to-shoot translocation of ^67^Zn in the WT. The results of this study suggest that Si accumulated in the shoot suppresses Zn through down-regulation of *OsZIP1*, the encoding transporter involved in Zn uptake (see Table 1), rather than by directly alleviating symptoms of Zn toxicity (e.g., oxidative tissue damage) as observed in the study of Song et al. (2011).

Greger et al. (2018) also reported that Si decreased Zn net accumulation in several plant species [(i.e., maize, lettuce (*Lactuca sativa*), wheat, carrot (*Daucus carota*) and pea (*Pisum sativum*)] even with adequate Zn supply, although the root Zn concentration increased. The authors therefore conclude that the binding of Zn to Si in the roots prevents Zn translocation to the shoot. But this poses another question which urgently needs an answer. Does Si application under low Zn conditions induce Zn deficiency?

### Manganese

Greger et al. (2018) showed that Si applied to soil increases manganese (Mn) availability and promotes Mn uptake and translocation to shoots in various plant species grown under conditions of adequate Mn supply. Only two studies, however, reported Si-mediated alleviation of Mn-deficiency (Bityutskii et al., 2014; de Oliveira et al., 2019). In cucumber, Si addition partly alleviated Mn-deficiency, possibly by indirectly reducing oxidative stress without increasing Mn transport and tissue accumulation (Bityutskii et al., 2014). Similarly, de Oliveira et al. (2019) showed that Si mitigates the effects of oxidative stress induced by Mn deficiency in sorghum plants by regulating the physiology and activity of antioxidative enzymes. Accumulation of Mn as a consequence of Si application was not observed. Considered together, these findings suggest that for plants inadequately supplied with Mn, Si supply plays an indirect role of improving antioxidant performance in mitigating the symptoms of Mn deficiency induced by ROS formation, rather than a direct one of increasing uptake and/or remobilization of Mn.

Interactions between Si and Mn in rice plants subjected to high Mn have been mainly attributed to decreased Mn accumulation in shoots (Horiguchi, 1988; Li et al., 2012). Decrease in Mn uptake can be explained by enhanced Mn oxidizing capacity of the rice roots, resulting in oxidation of plant-available Mn^2+^ on the root surfaces (Okuda and Takahashi, 1962). Che et al. (2016) showed Si-decreased Mn accumulation in rice shoots by decreasing root-to-shoot translocation of Mn, Mn-Si complex formation in root cells, and down-regulation of Mn-transporter gene *OsNramp5*. Enhanced Mn-tolerance was stimulated by Si in both Mn-tolerant and Mn-sensitive rice genotypes (Li et al., 2012). In the sensitive genotypes Mn increased in roots but not in shoots, whereas in the tolerant cultivars Mn was lower in both roots and shoots (Li et al., 2012). Indirectly, Si also enhanced Mn tolerance in rice by increasing synthesis of chlorophyll and ATP molecules and by stabilizing the structure of photosystem I (PSI) impaired by toxic Mn, which resulted in increased CO_2_ assimilation (Li et al., 2015). The exact mechanisms controlling Si-increased Mn tolerance in rice still remain unknown.

By contrast to rice, in many other plant species Si-induced Mn tolerance was attributed to altering Mn distribution within the plant, rather than by reducing Mn uptake (e.g., Dragisic-Maksimovic et al., 2012; Li et al., 2012; Che et al., 2016). For example, Si increased Mn-tolerance in pumpkin by localized accumulation of Mn in a metabolically inactive form at the base of trichomes of the leaf surface (Iwasaki and Matsumura, 1999). Conversely Si application resulted in homogenous distribution of Mn in the leaves of barley and common bean (Williams and Vlamis, 1957; Horiguchi and Morita, 1987). In maize, Si alleviates Mn toxicity by increasing the thickness of the epidermal layers, suggesting that Mn storage in non-photosynthetic tissue could be a Mn tolerance mechanism in this C4 crop (Doncheva et al., 2009). Iwasaki et al. (2002) also showed that total shoot Mn concentration was unaffected by Si supply. In cucumber, Si increased binding capacity of the cell wall to Mn, thereby lowering Mn concentration within the symplasm (Rogalla and Römheld, 2002a) and decreasing the free leaf apoplasmic Mn^2+^ as a catalyst for the Fenton reaction (Dragisic-Maksimovic et al., 2012). In support of these findings, Blamey et al. (2018) demonstrated that Si decreases Mn toxicity symptoms in cowpea, soybean and sunflower through increased Mn localization in leaf tissues by directly increasing apoplastic sequestration of Mn, in a nontoxic form, thereby decreasing apoplastic Mn^2+^ and excess Mn accumulation in the cytoplasm or apoplast. High Mn concentrations may have inhibited photosynthesis through several mechanisms, including suppressing chlorophyll and ATP synthesis, decreasing light-harvesting processes, impairing (PSI) stability and structure, and slowing activity of phosphoribulokinase.

### Copper

So far, interactions of Si with copper (Cu) have been studied in the context of Cu excess. Addition of Si relieved symptoms of Cu-toxicity such as chlorosis and reduction of the shoot and root biomass in wheat (Nowakowski and Nowakowska, 1997) and *Arabidopsis thaliana* (Li J. et al., 2008; Khandekar and Leisner, 2011). In general, Si-mediated alleviation of Cu-toxicity is mostly attributed to the immobilization of toxic Cu ions by enhancement of the cell wall binding capacity and the synthesis of Cu-binding molecules, both in roots and shoots. Although Si decreased the expression levels of two Cu transporter genes, *AtCOPT1* and *AtHMA5* in Arabidopsis roots (Table 1), it did not cause any changes in Cu status in leaves (Li J. et al., 2008). The authors explained that Si deposits formed in the cell walls increased Cu-binding sites and thus decreased impact of high Cu level in plant cells (Li J. et al., 2008). Furthermore, the expression of metallothioneins (MTs), the Cu-binding molecules, was maintained at high levels or is even increased in Si-treated plants suggesting that Si may also contribute to regulation of intracellular homeostasis of Cu, besides extending the additional cell wall binding sites for Cu (Khandekar and Leisner, 2011). However, Keller et al. (2015) found that Si induces Cu accumulation in the root epidermal cells, thus limiting root-to-shoot Cu translocation wheat (*Triticum turgidum*) seedlings. The author proposed a three-step mechanism: (1) increased Cu adsorption onto the outer thin layer root surface and immobilization in the vicinity of root epidermis; (2) increased Cu complexation by both inorganic and organic anions such as aconitate; and (3) limitation of translocation through the thickened Si-loaded endodermis areas. In bamboo (*Phyllostachys fastuosa*), Si also increased the concentration of S-ligands that chelate Cu^+^ into a less toxic form (Collin et al., 2014). Mateos-Naranjo et al. (2015) found that Si plays an important role in the Cu-tolerance of *Spartina densiflora* by reducing the excessive Cu translocation from roots to leaves.

Carrasco-Gil et al. (2018) demonstrated that Si application causes higher Fe plaque formation on the root surface of rice which significantly increased Cu concentration, probably as a consequence of a Fe/Cu antagonism. Silicon reduces Cu toxicity symptoms in *Erica andevalensis* by decreasing translocation of Cu from roots to shoots, and also by immobilizing or deactivating Cu in the Si phytoliths in leaves (Oliva et al., 2011). Recently, Bosnic et al. (2019a) provided evidence that binding of Cu to the Cu-chelating proteins, such as Zn/Cu SOD in roots and plastocyanin in leaves, are important components of the Si-alleviating mechanism in cucumber exposed to Cu excess. In cucumber, Si supply also enhanced the Cu binding capacity of the root cell wall, as well as the accumulation of Cu-ligands such as organic acids (citrate and malate in the roots and aconitate in the leaves) and amino acids (nicotianamine and histidine) in leaves (Bosnic et al., 2019a,b). Flora et al. (2019) suggested that Si-mediated alleviation of Cu toxicity in tobacco (*Nicotiana tabacum*) by decreasing root uptake of Cu, also down-regulates expression of *NtCOPT1* (Table 1), and elevates expression of genes involved in biosynthesis of ethylene. Similarly, Kim et al. (2014) suggested that a higher accumulation of Si in the roots of Cu-stressed rice plants in turn reduced influx of Cu by down-regulation of metal transporter genes *OsHMA2* and *OsHMA3* (Table 1).

### Boron

The beneficial effect of Si on boron (B) disorders (deficiency and excess) has been reported for both dicotyledonous and graminaceous species and several mechanisms have been proposed by which Si can alter B uptake and transport. Both elements show considerable chemical similarity as for example in the presence of weak undissociated acids in aqueous solution and affinity for binding to polyhydroxy compounds (Brown et al., 1999; Kinrade et al., 1999). Moreover, both elements are taken up as uncharged acids (H_4_SiO_4_^0^, pKa of 9.8; H_3_BO_3_^0^, pKa of 9.2) either actively or passively depending on their external concentrations. Under certain environmental conditions it is therefore possible that Si complexes with B in soil solution thus reducing availability of B and its potential phytotoxic effect (Nozawa et al., 2018). Although ameliorative effect of Si via decreased B accumulation has been reported in numerous species, i.e., tomato (Kaya et al., 2011), grapevine rootstocks (*Vitis* sp.) (Soylemezoglu et al., 2009) and spinach (*Spinacia oleracea*) (Gunes et al., 2007), the mechanism of Si-mediated B uptake and/or transport still remains purely speculative. According to Rogalla and Römheld (2002b), application of Si to cucumber exposed to high B had no effect on total B concentration, but strongly affected B compartmentation within leaves; a higher B concentration was found in the cell wall fraction than in the press sap of leaves of Si-treated cucumber plants.

In barley, Akcay and Erkan (2016) showed that simultaneous application of B and Si increased the transcript level of the gene encoding the BOR2 efflux transporter (see Table 1), responsible for B detoxification in the apoplast. Interestingly, the authors reported a higher transcript response in the shoot compared to the root which could explain the effective prevention of B accumulation and increased tolerance to high B (Miwa and Fujiwara, 2010). Accordingly, Akcay and Erkan (2016) reported the existence of a certain degree of competition within the B transport system favoring Si uptake, which was also the mechanism proposed for oilseed rape grown in excessive B (Liang and Shen, 1994). Indeed, in both barley and rice, B can be transported through the multifunctional HvNIP2;1 transporter (homolog of OsLsi1) which has been demonstrated in oocyte experiments (Schnurbusch et al., 2010; Mitani-Ueno et al., 2011). However, in barley genotypes differing in susceptibility to B toxicity, no competitive interaction was found in the uptake of Si and B (Nable et al., 1990).

### Chlorine and Nickel

In saline soils, high concentrations of both sodium (Na) and chlorine (Cl) are equally phytotoxic. Most studies regarding the beneficial effect of Si under saline conditions have dealt with uptake, exclusion and compartmentation of Na. It is for this reason that the effect of Si on Cl transport and accumulation under saline condition is much less well understood compared to Na (see “Sodium” subsection, below). In a study using three rice cultivars differing in salt tolerance, Shi et al. (2013) demonstrated that Si improved growth of all three cultivars and markedly decreased shoot but not root Cl^–^ concentration. Moreover, Si supply increased transpiration rate, net photosynthetic rate, and stomatal conductance in plants exposed to salinity. The authors suggest, however, that Si-mediated alleviation of Cl toxicity was mainly attributed to the reduction in root-to-shoot transport (Shi et al., 2013). In addition, some authors suggest that Cl^–^ recirculation from shoot to root via the phloem could offer an efficient mechanism in controlling Cl^–^ accumulation in the plant shoot (White and Broadley, 2001). Liang and Ding (2002) reported lower Cl^–^ accumulation in the epidermal, cortical and stellar cells of barley roots subjected to salt stress. Moreover, Si markedly decreased Cl concentration in various plant organs, such as shoots of tomato and spinach (Gunes et al., 2007), root of grapevine (Soylemezoglu et al., 2009), and root, stem, and leaves of aloe (Xu et al., 2015). However, the mechanism by which Si affects Cl transport, distribution and its accumulation in plant tissues remains to be elucidated.

It appears that only two studies have reported Si-mediated regulation of Ni uptake and accumulation in plants. It has been shown that Si addition decreased Ni concentrations in both roots and leaves of mustard (*Brassica juncea*) and cotton plants, thus alleviating the inhibitory effect of Ni stress on biomass production (Khaliq et al., 2016; Abd_Allah et al., 2019). The reason for reduced Ni uptake, however, may possibly be that of decreased soil Ni availability due to the increase in pH of soil fertilized with sodium metasilicate, rather than a Si-alleviating effect *per se* (Weng et al., 2003).

## Interactions of Silicon With Beneficial Elements

### Aluminum

Numerous studies, have shown that Si supply affects aluminum (Al) uptake, and subsequently plant tolerance to Al-toxicity, by either external (*ex planta*) and/or internal (*in planta*) mechanisms (see Hodson and Evans, 2020 and reference therein). Early investigations showed that the ameliorative effect of Si on Al toxicity could mainly be attributed to increased pH of the rhizosphere. Likewise in a hydroponic study with sorghum, Li et al. (1996) reported that Si-induced alleviation of Al toxicity was due to increase in pH and not to the direct effects of Si on Al in nutrient solution. From chemical studies on Al-Si interaction in solution it was also known that pH influenced the formation of sub-colloidal inert hydroxyaluminosilicate species (HAS) (Exley and Birchall, 1992, 1993). However, the potential impact of HAS on plant growth was not appreciated until Baylis et al. (1994) reported that at low pH, a higher concentration Si was required to ameliorate Al toxicity by soybean; the authors postulated that basis for this higher demand for Si was the higher affinity of Si for Al in HAS formation at low pH (see Hodson and Evans, 2020). The beneficial effect of silicic acid was also confirmed in later studies on Al-Si interaction in maize (Ma et al., 1997). Interaction between Al and Si in the root apoplast is considered as a complex process governed by a range of interacting factors, such as apoplastic pH and concentrations of Al and Si. Accordingly, Rowatt and Williams (1996) suggested that Si added to maize, buffered H^+^ in the root apoplast which increased pH at the root surface, and thereby induced HAS formation. Similarly, Cocker et al. (1998) proposed the Si-induced formation of nontoxic HAS in the root apoplast of wheat as a more likely mechanism for controlling Al availability.

In addition to HAS formation, Corrales et al. (1997) suggested that decreased Al uptake can be due to enhanced root exudates that precipitate Al. In this study, maize plants pre-treated with Si showed more hematoxylin stainable precipitates at the root surface compared to the -Si control. Colocalization of Si and Al was shown in the epidermal cells of sorghum roots (Hodson and Sangster, 1993) and in epidermal and hypodermal cells of wheat roots (Cocker et al., 1997). These authors hypothesized that colocalization of Si and Al may, to some extent, slow down Al transport from entering the inner root cortex. Recently, Kopittke et al. (2017) confirmed this hypothesis by clearly demonstrating colocalization of Al and Si in both mucigel and outer apoplast of the root apex of sorghum. Besides grasses, Si and Al codeposition in roots cortex cell walls has also been confirmed in woody species such Norway spruce (*Picea abies* L.) (Hodson and Wilkins, 1991; Prabagar et al., 2011). Likewise, in a study on *Faramea marginata* (Rubiaceae), considered as an Al accumulator, Britez et al. (2002) provided first evidence for Al-Si complexation, in plant shoot tissue. Since most species considered as Al accumulators use organic ligands (Hodson and Evans, 2020) for Al complexation, this work was first to show the complexation role of inorganic ligands. Similarly, in a study conducted on another member of Rubiaceae (*Rudgea viburnoides*), Malta et al. (2016) reported Al and Si co-localization mostly in the epidermis of roots, stems, and leaves.

Moreover, an interesting study by Feng et al. (2019) involving the root border cells of pea, showed that these cells were coated with extracellular silica layers, and therefore were able to adsorb Al on their surfaces preventing Al transport into the cells. Very recently, Xiao et al. (2021) reported involvement of Si in decreasing Al accumulation (i.e., non exchangeable Al fraction) in the cell wall of rice root apex by decreasing hemicellulose content, which is a main binding site for Al in the cell wall without affecting its cation exchange capacity.

### Sodium

Extensive studies have shown that precipitated amorphous Si deposits can fortify the root apoplast and cell wall surrounding the vasculature (Gong et al., 2006). Si can therefore act as a mechanical barrier for root-to-shoot translocation of Na^+^ via the apoplastic bypass route (Yeo et al., 1999), thus preventing accumulation of Na^+^ in the shoot. However, bypass flow of Na^+^ in response to Si in rice has been shown to be cultivar dependent (Yeo et al., 1999; Flam-Shepherd et al., 2018). Very recently, Yan et al. (2021a) investigated the role of Si in bypass flow and root-to-shoot translocation of Na^+^ in rice using *lsi1* and *lsi2* mutants defective in OsLsi1 and OsLsi2, respectively, and their wild types (WTs). Under salt stress, Si promoted plant growth and decreased root-to-shoot Na^+^ translocation in WTs but not in mutants. The authors concluded that Si-induced Na^+^ bypass flow is a spatially dependent process which occurs mainly in the root endodermis. Besides acting directly as the apoplastic bypass route blocker, Si supply can also enhance lignification and suberization of the Casparian band (Fleck et al., 2015), by altering the expressions of genes related to phenol biosynthesis (Hinrichs et al., 2017), thus further protecting plants against uncontrolled Na^+^ influx. Although evidence of tissue Na^+^ complexation by Si is lacking, Si co-precipitation with Na^+^ in the extracellular matrix was reported for wheat (Saqib et al., 2008). Bosnic et al. (2019c) demonstrated that Si supply increases binding capacity of cell walls of maize roots for Na^+^, thereby decreasing free Na^+^ for transport through the plasma membrane. Considering that influx of Na^+^ is mediated by various transporters some of which are sensitive to Ca^2+^ (Davenport and Tester, 2000; Demidchik and Tester, 2002), interaction of Si and Ca in growing media can lead to increased Na accumulation in roots (Dishon et al., 2011; Bosnic et al., 2019c).

Bosnic et al. (2018) demonstrated that Si supply to moderately NaCl-stressed maize plants reduced Na concentration in the root symplast by up-regulating the expression of *SOS1* (responsible for Na^+^ efflux) and down-regulating the expression of *HKT1* (responsible for Na^+^ influx). These authors also demonstrated higher sequestration of Na^+^ in the leaf vacuoles and concomitantly lower Na^+^ concentrations in chloroplasts. This effect can be partially attributed to Si-mediated increase of both H^+^-ATPase and H^+^-PPase activities (Liang, 1999; Liang et al., 2005; Xu et al., 2015). However, a direct link between enzyme activities and the potential to generate a motive force for H^+^/Na^+^ antiport for both plasma membrane and tonoplast still remains speculative.

Flam-Shepherd et al. (2018) tested the effects of Si in mediating ^24^Na^+^ membrane flux regulation in two hydroponically grown rice cultivars, one salt-sensitive (IR29) and the other salt-tolerant (Pokkali). Si had no effect on Na^+^ influx or efflux or on Na^+^ stimulated plasma membrane depolarization. However, it increased growth and lowered Na in shoots of both cultivars. In the salt sensitive cultivar IR29, Si lowered shoot Na by a large reduction in bypass flow whereas in cv. Pokkali where bypass flow was small, the lower Na resulted from growth dilution. The authors suggest that additionally other Si triggered processes are likely to underlie the improved growth of the two cultivars including changes in gene expression (Debona et al., 2017). The study also casts doubt on possible influence of Si on Na^+^ transport across cell membranes into and out of roots.

### Selenium

Selenium (Se) is an essential nutrient in the human diet and supplied to nearly half the world‘s population by the staple rice crop, a Si accumulator, which is however low in Se (Zhou et al., 2020). The interaction between Se and Si is thus of high importance. Two main plant-available Se inorganic forms *viz.* selenite and selenate are readily taken up by plant roots via both active and passive transport systems (Li H.-F. et al., 2008; Lazard et al., 2010). In rice, selenite, the main bioavailable Se form in paddy soils, uses the same root influx transporter (OsNIP2;1, known as OsLSi) as silicic acid (Zhao et al., 2010). These authors showed a significant decrease in the selenite concentration in both xylem sap and shoots in *lsi1* rice mutant, but not in *lsi2* mutant defective in Lsi2 efflux transporter (Zhao et al., 2010). Furthermore, they hypothesized that in the *lsi1* mutant the absence of an antagonistic effect from silicic acid could be due to specific metabolic processes of Se in root cells following uptake with such Se-organo-complexes being loaded into the xylem via efflux transporters other than Lsi2.

In tomato, addition of a high Si dose (1 mM) significantly decreased root concentration of Se at pH 3, but had no effect at higher (up to 8) pH (Wang et al., 2019). Such pH-dependent disparity on Si effect could partially be explained by Si-influx transporter affinity for Se form available at the given pH. Moreover, the presence of the fully operative Si influx transporter (SlLsi1), and the lack of functional Si efflux transporter (SlLsi2; involved in xylem loading of Si) in tomato (Sun et al., 2020), could also explain the lower Se accumulation in the tomato roots as a consequence of Si/Se uptake antagonism, as well as lack of Si effect on Se accumulation in the shoot reported by Wang et al. (2019).

## Essential and Beneficial Element Status Affects Silicon Uptake and Distribution

To date, two different Si transporters, Lsi (channels) and Lsi2 (anion-type transporter) have been characterized in the roots of various plant species including rice, barley, maize, wheat, soybean, pumpkin, cucumber, tobacco, sorghum, tomato (for recent review see Mitani-Ueno and Ma, 2020), and recently date palm (*Phoenix dactylifera*) (Bokor et al., 2019) and grapevine (Noronha et al., 2020) have been added to the list. All these species differ greatly in ability to accumulate Si in upper plant parts. Silicon in the form of H_4_SiO_4_ enters the roots via influx aquaporin channels belonging to the Nodulin 26-like intrinsic proteins (NIPs) and is then, exported out of the endodermis as H_4_SiO_3_^–^ via the efflux anion H^+^/antiporter Lsi2, and loaded into the xylem. However, the expression pattern, cell-type-specific expression, polar localization and functionality of these transporters differ among plant species. For instance, tomato which shows very low shoot accumulation of Si, has the functional influx transporter SlLsi1 for root uptake of Si, but not the functional efflux transporter SlLsi2 for its upward transport (Sun et al., 2020). Once loaded into the xylem, Si is moved upward to the shoot by the transpiration stream, and is finally unloaded from the xylem vessels via Lsi6 (Mandlik et al., 2020). Lsi6 is an influx channel homolog of Lsi1, which shows polar localization in the xylem parenchyma cells. As yet, Lsi6 has been characterized only in rice, barley and maize.

Whether nutrition imbalance (deficiency or excess) can influence root uptake, root-to-shoot transport and tissue distribution of Si is still unknown. Resolving this puzzle is additionally complicated due to a lack of overall understanding of the Si uptake/transport system in the whole plant. Factors affecting the regulation of the Si-transport systems are almost completely unknown. So far, it has been demonstrated that Si down-regulates influx and efflux Si transporters (OsLsi1 and OsLs2) in rice (Mitani-Ueno et al., 2016). By contrast, in maize and barley, only efflux transporters (Lsi2) are down-regulated by Si (Mitani et al., 2009), while influx transporters Lsi1 are unaffected (Chiba et al., 2009). According to available literature, environmental factors such as drought or herbivore attack can also modulate Si uptake (e.g., Reynolds et al., 2009; Yamaji and Ma, 2011).

Increasing evidence suggests that nutritional status can affect Si accumulation and distribution in plants (e.g., Kim et al., 2014; Bokor et al., 2015; Hosseini et al., 2017; Wu et al., 2017; Chaiwong et al., 2020; Minden et al., 2020; Xiao et al., 2021). However, only a few studies have investigated the effect of specific nutrient imbalances on the expression of Si transporters and could be additionally affected by the Si concentration in the shoot (Mitani-Ueno and Ma, 2020). It has been shown that limited supply of the macronutrients (N, P, K) and the micronutrient Fe, can induce Si accumulation in plant roots and/or shoots (Wu et al., 2017; Chaiwong et al., 2018, 2020; de Tombeur et al., 2020; Minden et al., 2020). Exudation of organic ligands (mainly PSs) by roots of Fe deficient barley plants can indirectly increase Si availability in the rhizosphere (Gattullo et al., 2016), as a consequence of nutrient (e.g., Fe) mobilization from silicate minerals by liberation of silicic acid. In rice roots, low supply of both N and Fe led to up-regulation of *OsLsi1* (Wu et al., 2017; Chaiwong et al., 2020) and *OsLsi2* (Wu et al., 2017). A similar effect was observed in K-deficient barley, where up-regulation of *HvLsi1*, *HvLsi2* and *HvLsi6* was responsible for accumulation of Si in the shoot (Hosseini et al., 2017). Moreover, N and P starvation influenced not only uptake but also distribution of Si; under P deficiency, the grass (*Holcus lanatus*) tended to accumulate Si in aboveground parts, whereas in N deficient plants of the same species, Si was accumulated in the roots (Minden et al., 2020). Under optimal growing conditions, however, Si was evenly distributed between shoot and roots. At a whole plant level, P-deficient plants take up more Si than those subjected to N deficiency (Minden et al., 2020).

On the other hand, exposure of plants to excessive supply of mineral nutrients also modulates expression of Si transporters, however, the effect (up- or down-regulation) seems to be dependent not only on plant species, but also on specific nutrient conditions. High N and Zn supply reduced transcript levels of *Lsi1* and *Lsi2* in rice and maize, thus limiting Si uptake and, consequently, decreasing Si content in roots and/or leaves (Bokor et al., 2015; Wu et al., 2017). Interestingly, in maize, *ZmLsi6* was up-regulated (Bokor et al., 2015), potentially indicating Si re-translocation as a response to metal toxicity. In addition to the previously mentioned studies, several reports also showed that high N supply limits Si accumulation in various plant species such as wheat, rice and *Brachypodium distachyon* (e.g., Wu et al., 2017; Głazowska, 2018; Deus et al., 2020). The negative effect of high N supply on Si accumulation was confirmed in *Brachypodium distachyon* by comparing Si accumulation between WT and *Bdls1-1*, mutant defective in influx Si transporter Lsi1 (Głazowska, 2018). By contrast, high Cu or Al resulted in higher Si accumulation in tobacco, rice and ryegrass roots and/or leaves (Kim et al., 2014; Pontigo et al., 2017; Flora et al., 2019; Xiao et al., 2021), as a result of the increased expression of *Lsi1* and *Lsi2* (see Table 2).

**TABLE 2:** Evidence for expression of Si transporter genes affected by supply of essential and beneficial elements.

Conditions	Plant species	Localization/direction/form/function	Si transporter genes	Expression pattern affected by element supply	References
Low N	Rice	Root/influx/H_4_SiO_4_/uptake	*OsLsi1*	Up-regulated	Wu et al., 2017
		Root/efflux/H_4_SiO_3_^–^/xylem loading	*OsLsi2*	Up-regulated	
High N	Rice	Root/influx/H_4_SiO_4_/uptake	*OsLsi1*	Down-regulated	Wu et al., 2017
		Root/efflux//H_4_SiO_3_^–^/xylem loading	*OsLsi2*	Down-regulated	
Low K	Barley	Root/influx/H_4_SiO_4_/uptake	*HvLsi1*	Up-regulated	Hosseini et al., 2017
		Root/efflux//H_4_SiO_3_^–^/xylem loading	*HvLsi2*	Up-regulated	
		Leaf/influx/H_4_SiO_4_/xylem unloading	*HvLsi6*	Up-regulated	
Low Fe	Rice	Root/influx/H_4_SiO_4_/uptake	*OsLsi1*	Up-regulated	Chaiwong et al., 2020
High Cu	Rice	Root/influx/H_4_SiO_4_/uptake	*OsLsi1*	Up-regulated	Kim et al., 2014
		Root/efflux//H_4_SiO_3_^–^/xylem loading	*OsLsi2*	Up-regulated	
High Zn	Maize	Root/influx/H_4_SiO_4_/uptake	*ZmLsi1*	Down-regulated	Bokor et al., 2015
		Root/efflux//H_4_SiO_3_^–^/xylem loading	*ZmLsi2*	Down-regulated	
		Leaf/influx/H_4_SiO_4_/xylem unloading	*ZmLsi6*	Up-regulated	
High Al	Rice	Root/influx/H_4_SiO_4_/uptake	*OsLsi1*	Up-regulated	Xiao et al., 2021
		Root/efflux//H_4_SiO_3_^–^/xylem loading	*OsLsi2*	Up-regulated	
	Ryegrass	Root/influx/H_4_SiO_4_/uptake	*LpLsi1*	Up-regulated	Pontigo et al., 2017
		Root/efflux//H_4_SiO_3_^–^/xylem loading	*LpLsi2*	Up-regulated	

## Conclusion and Perspectives

**FIGURE 2:**
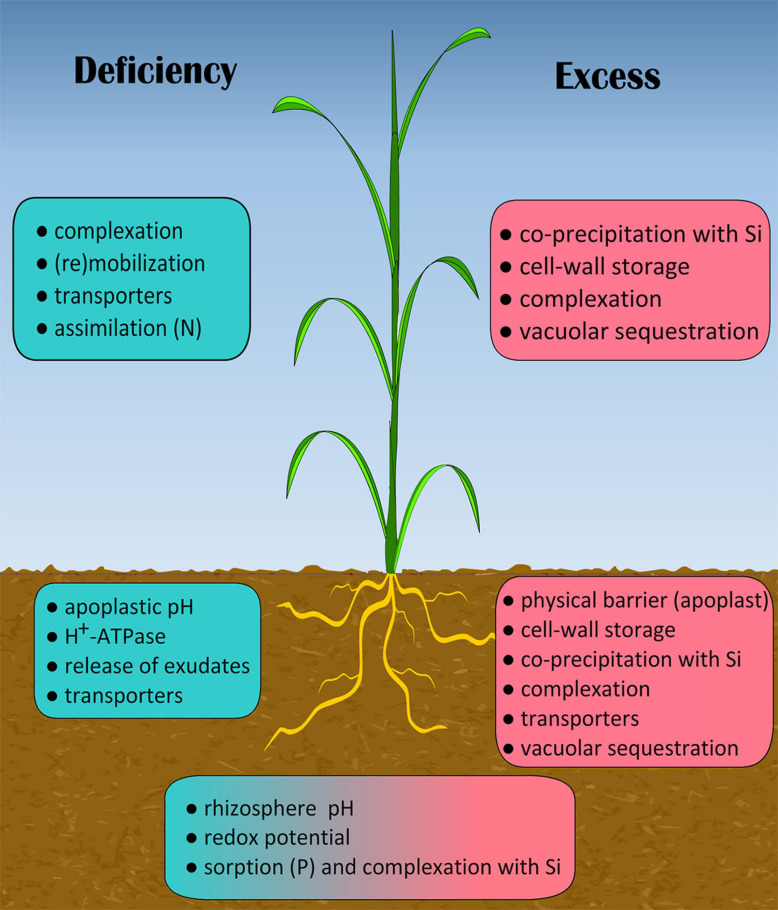
Processes directly involved in rhizosphere mobilization, root uptake, root-to-shot transport and shoot utilization of essential (nutrients) and beneficial elements modulated by Si. Left (blue), nutrient deficiency; right (pink), excess of nutrients and beneficial elements; middle (blue/pink), nutrient deficiency or excess of nutrients and beneficial elements. General ameliorating effect of Si in stressed plants (e.g., increased tissue antioxidant capacity) may also improve overall plant performance thereby enhancing the above-mentioned processes.

From this review, it is clear that interactions of Si with essential (nutrients) and beneficial mineral elements are largely plant-specific and also dependent on mineral status (deficiency or excess). These interactions firstly occur in the rhizosphere and in the root apoplast which include mobilization of nutrients from the rhizosphere by root exudates under low nutrient conditions and by buffering soil and cell wall availability under excess (Figure 2). Moreover, Si is able to control the availability of different elements in soils by competing for binding on soil particles depending on speciation of silicic acid (for review see Schaller et al., 2021). In the context of root uptake and transport (including remobilization) of nutrients, Si supply affects the expression of transporter genes differently for several mineral elements (Table 1), although the use of gene expression as a proxy for change in transport protein abundance or activity needs to be treated with caution (Coskun et al., 2019). On the other hand, nutrient status and excess of some beneficial elements may alter expression of Si transporter genes (*Lsi1*, *Lsi2*, *Lsi6*) thereby changing uptake and distribution of Si in plants (Table 2). The effectiveness of Si on overall plant performance depends on both Si and nutrient tissue concentrations, so that the existence of a feed-back loop between Si and nutrients is self-evident although the nature of this cross-linkage is unclear.

The role of Si in modulating nutrient acquisition and utilization appears to be more complex (Figure 2), and thus the possibility of a signaling function of Si in transcriptional regulation of genes responsible for root acquisition and tissue retranslocation cannot be disregarded. Clearly, Si is involved in a variety of mechanisms in regulating nutrient deficiency and toxicity in different plant species. Therefore, a comprehensive understanding of the role of Si in regulation of uptake and transport systems for nutrients and other mineral elements (including beneficial elements) and *vice versa* is urgently needed. In particular, we are far from fully understanding the molecular mechanisms of Si-mediated nutrient transport which may explain the wide diversity among plant species.

In this respect, a deeper understanding of exactly how Si regulates transcription of transporter genes will be of benefit in improving crop productivity, yield quality and food safety. Also, detailed knowledge of the specific role of nutrients in mobilization from rhizosphere to uptake and transport of Si is essential for increasing plant resistance to both biotic and abiotic stresses. We hope that this review will also foster new research in the study of interactions between Si and nutrients of relevance to improving use efficiency of mineral fertilizers and pesticides in agricultural practice.

## Author Contributions

MN conceived this review. JP, LK, PB, and MN wrote the draft, and designed the figures and tables. MN and EAK reviewed the manuscript. All authors approved the final version.

## Conflict of Interest

The authors declare that the research was conducted in the absence of any commercial or financial relationships that could be construed as a potential conflict of interest.
